# Prognostic and immunological role of CD36: A pan-cancer analysis

**DOI:** 10.7150/jca.50502

**Published:** 2021-06-11

**Authors:** Yong-Jian Chen, Wei-Xin Liao, Shao-Zhuo Huang, Yun-Fang Yu, Jing-Yun Wen, Jie Chen, Da-Gui Lin, Xiang-Yuan Wu, Nan Jiang, Xing Li

**Affiliations:** 1 Department of Medical Oncology, The Third Affiliated Hospital of Sun Yat-sen University, Guangzhou, China.; 2 Department of Infectious Diseases, The Third Affiliated Hospital of Sun Yat-sen University, Guangzhou, China.; 3 Department of General Surgery, The Third Affiliated Hospital of Sun Yat-sen University, Guangzhou, China.; 4 Department of Medical Oncology, Sun Yat-sen Memorial Hospital, Sun Yat-sen University, Guangzhou, China.; 5 Department of Colorectal Surgery, Sun Yat-sen University Cancer Center, Guangzhou, China.; 6 Department of Transplantation, the Second Affiliated Hospital of Southern University of Science and Technology and the Third People's Hospital of Shenzhen, Shenzhen, China.

**Keywords:** CD36, Biomarker, Immunotherapy, Pan-cancer analysis

## Abstract

CD36 plays a critical role in lipid metabolism, which is closely associated with human immunity. However, the role of CD36 in cancer remains unclear. We performed a pan-cancer analysis to elucidate the potential role of CD36 in cancer by investigating its prognostic value and current predictors for the efficacy of immune checkpoint inhibitors (ICIs) in multiple cancer types. CD36 expression in cancer cell lines, tumor tissue, and their adjacent normal tissues displayed heterogeneity among different cancers. Immunohistochemistry was used to detect CD36 expression and confirmed the results. CD36 expression significantly affects prognosis in the six cancer types. High CD36 expression was marginally associated with poorer prognosis in four of them and improved prognosis in the remaining two types. CD36 expression was significantly correlated with the 6 immune infiltrates in most cancer types. In addition, CD36 gene expression was positively correlated with Stromal score, Immune score, and ESTIMATE score. A total of 47 immune checkpoint genes were collected and their relationship with CD36 expression was analyzed. CD36 expression was significantly associated with multiple stimulatory and inhibitory checkpoint molecules with a disease-specific pattern. As to the genes reported to positively relate to the efficacy of ICIs, CD36 expression was positively correlated with most of them but negatively associated with a small proportion of cancer type-specific patterns. Concerning the genes negatively related to the efficacy of ICIs, CD36 expression was positively correlated with NRP1 and TNFSF15 in multiple cancers. CD36 expression was negatively correlated with tumor neoantigen burden in most cancer types. However, CD36 expression was negatively correlated with tumor mutation burden in most cancer types. The correlation between CD36 expression and the four methyltransferases was also significant in multiple cancers, but also with a cancer type-specific pattern. In summary, the current study found CD36 expression and its prognostic value in multiple cancer types. In addition, the expression of CD36 was significantly associated with current predictors for the efficacy of ICIs. The practical application value of CD36 is disease specific.

## Introduction

Immune checkpoint inhibitors (ICIs) have revolutionized the therapeutic modalities for all types of cancers. Anti-cytotoxic T-lymphocyte antigen-4 (CTLA-4) and anti-programmed death-1 (PD-1)/PD-L1 therapies have achieved significant success in treating numerous cancers. However, only a proportion of patients respond to these therapies. Although the safety profile of ICI treatment was much better than that of traditional chemotherapy, severe immune-related adverse events (irAEs) among patients undergoing ICI therapy could have occurred from the first administration to more than 6 months after initial treatment, which was not as easy to predict as that of chemotherapy. Thus, it is imperative to identify prognostic factors for the efficacy or toxicity of ICI treatment. Multiple prognostic systems have been built for ICI treatment, including immune cells, PD-L1 overexpression, neoantigens, and genetic and epigenetic signatures. However, none have achieved satisfactory accuracy.

Lipid metabolism has been reported to be a key regulator of cancer immunology. CD36 is a scavenger receptor expressed in a variety of cell types. In recent years, researchers have found that CD36 is a multiligand receptor protein that binds to a variety of ligands, including apoptotic cells, thrombospondin-1 (TSP-1), and fatty acids (FAs) 1. Its biological functions involve lipid uptake, immune recognition, inflammation, molecular adhesion and apoptosis, inflammatory response, apoptosis phagocytosis, angiogenesis, energy metabolism, and tumor metastasis 2-4. CD36 not only promotes tumor metastasis and treatment resistance by promoting lipid uptake and FA oxidation, but also inhibits angiogenesis by binding with TSP-1, thus inducing tumor microvascular endothelial cell apoptosis or blocking the vascular endothelial growth factor receptor 2 pathway 5-7. In addition, CD36-driven lipid metabolism reprogramming and the function of tumor-associated immune cells lead to tumor immune tolerance and tumorigenesis. Significant progress has been made in demonstrating regulatory networks that control the unique physiological characteristics of CD36, indicating that targeting CD36 is a potential cancer treatment strategy 8-11.

In this study, we performed a pan-cancer analysis to elucidate the potential role of CD36 in cancers by investigating its prognostic value and current predictors for the efficacy of ICIs in multiple cancer types.

## Methods

### Data processing

All included data were extracted from the Cancer Genome Atlas (TCGA), Cancer Cell Line Encyclopedia (CCLE), and the Genotype-Tissue Expression (GTEx) databases.

### Survival analysis

Survival curves were generated using Kaplan-Meier plots. The results are displayed as hazard ratios and *P*-values from a log-rank test. Kaplan-Meier curves with log-rank tests were used to determine survival differences.

### Tumor immune infiltration analysis

The abundance of immune infiltrates was estimated using a web server for comprehensive analysis of tumor infiltrating immune cells, named the Tumor Immune Estimation Resource (TIMER) database 12.

### ESTIMATE analysis

Immune and stromal scores were calculated using the ESTIMATE (Estimation of STromal and Immune cells in Malignant Tumor tissues with Expression data) algorithm. The ESTIMATE is an algorithm that provides scores for the level of stromal cells present and the infiltration level of immune cells in tumor tissues by calculating specific molecular biomarker expression in immune and stromal cells to predict the tumor microenvironment. The stromal score captures the presence of stroma, the immune score represents the infiltration of immune cells, and the ESTIMATE score infers tumor purity and is equal in number to stromal score and immune score 13.

### Immune checkpoint gene correlation analysis

We analyzed the correlation between CD36 and Immune checkpoint gene (ICG) expression, such as cytotoxic T-lymphocyte protein 4 (CTLA4), programmed cell death protein 1 (PDCD1), and programmed cell death 1 ligand 1 (CD274). The association of gene expression was evaluated using Spearman's correlation and statistical significance.

### Tumor neoantigen burden (TNB) and tumor mutational burden (TMB) calculations

The prediction of TNB was accomplished using NetMHCcons called from the Immune Epitope Database (IEDB). All samples met the minimum read depth requirements, and neoantigens with a predicted IC50>500 nM and a rank score of more than 2 were excluded 14. TMB is a measure of the number of mutations per megabyte in tumor tissue, and it is also the mutation density of tumor genes. It is defined as the average number of mutations in the tumor genome, including the total number of gene coding errors, base substitutions, and gene insertions or deletions. The estimated value of TMB for each sample was equal to the total mutation frequency/38 15.

### DNA methylation analysis

DNA methylation is the covalent bond of a methyl group to the cytosine 5 carbon position of the genomic CpG dinucleotide under the action of DNA methylation transferase. Here, we analyzed the correlation between gene expression and the expression of four methyltransferases (DNMT1: red, DNMT2: blue, DNMT3A: green, and DNMT3B: purple).

### Patients and healthy donors

Patients with kidney renal clear cell carcinoma (KIRC), liver hepatocellular carcinoma (LIHC), breast invasive carcinoma (BRCA), colon adenocarcinoma (COAD), esophageal carcinoma (ESCA), lung adenocarcinoma (LUAD), lung squamous cell carcinoma (LUSC), or thyroid carcinoma (THCA) who had undergone radical surgery at the Third Affiliated Hospital of Sun Yat-sen University and the Third People's Hospital of Shenzhen during the period between December 2018 and September 2020 were enrolled in this study retrospectively, with their paraffin-embedded tumor and para-tumor tissues analyzed using immunohistochemistry. The sample size was 10 for each cancer type. The diagnosis of the indicated cancer was confirmed based on pathological findings. All patients were also screened for the serum human immunodeficiency virus (HIV) antibody, hepatitis B surface antigen (HBsAg), hepatitis C virus antibody, hepatitis D virus (HDV) antigen, and HDV antibody. Patients and healthy controls who were positive for HIV or hepatitis virus infection, pregnant, or receiving anti-cancer therapies were excluded from this study. This study was approved by the Clinical Ethics Review Board of the Third People's Hospital of Shenzhen and the Third Affiliated Hospital of Sun Yat-sen University. Written informed consent was obtained from all patients at the time of admission.

### Immunohistochemistry analysis

The expression of CD36 in paraffin-embedded tumor and para-tumor tissues was analyzed by immunohistochemistry. Briefly, the 4-µm thick sections were dewaxed in fresh xylene and then hydrated with gradient ethanol. We used citrate buffer to repair antigens in a microwave oven. Hydrogen peroxide (3%) was used to block endogenous peroxidase. Then, the slices were incubated with the first antibody at 4 °C overnight. The second antibody was added and incubated at 32 °C for 30 min. The chromogenic 5 min was produced by Diaminobenzidine (DAB). Following hematoxylin re-dyeing for 1 min, gum seals were made after dehydration and transparency. Immunohistochemical staining was evaluated by two independent pathologists, without knowledge of the clinicopathological information of these patients. CD36 expression levels were evaluated by integrating the percentage of positive cells and staining intensity. The scoring criteria were as follows: (1) percentage of positive cells: no staining (score 0), 0%-25% (score 1), 26%-50% (score 2), 51%-75% (score 3), and 76%-100% (score 4); (2) staining intensity, negative (score 0), weak positive (score 1), positive (score 2), and strong positive (score 3). The final histochemistry score was produced by the product of the percentage of positive cells and the score of staining intensity.

### Statistical analysis

The results of the Kaplan-Meier plots are displayed as HRs and *P*-values from a log-rank test. The correlation of gene expression was evaluated using Spearman's correlation and statistical significance. The patient data we used were acquired from publicly available datasets that were collected with patients' informed consent. P-values <0.05 were considered statistically significant. Further analysis was conducted by Sangerbox (http://www.sangerbox.com/tool).

## Results

### CD36 expression in different types of tissue or cancer

**Figure 1:**
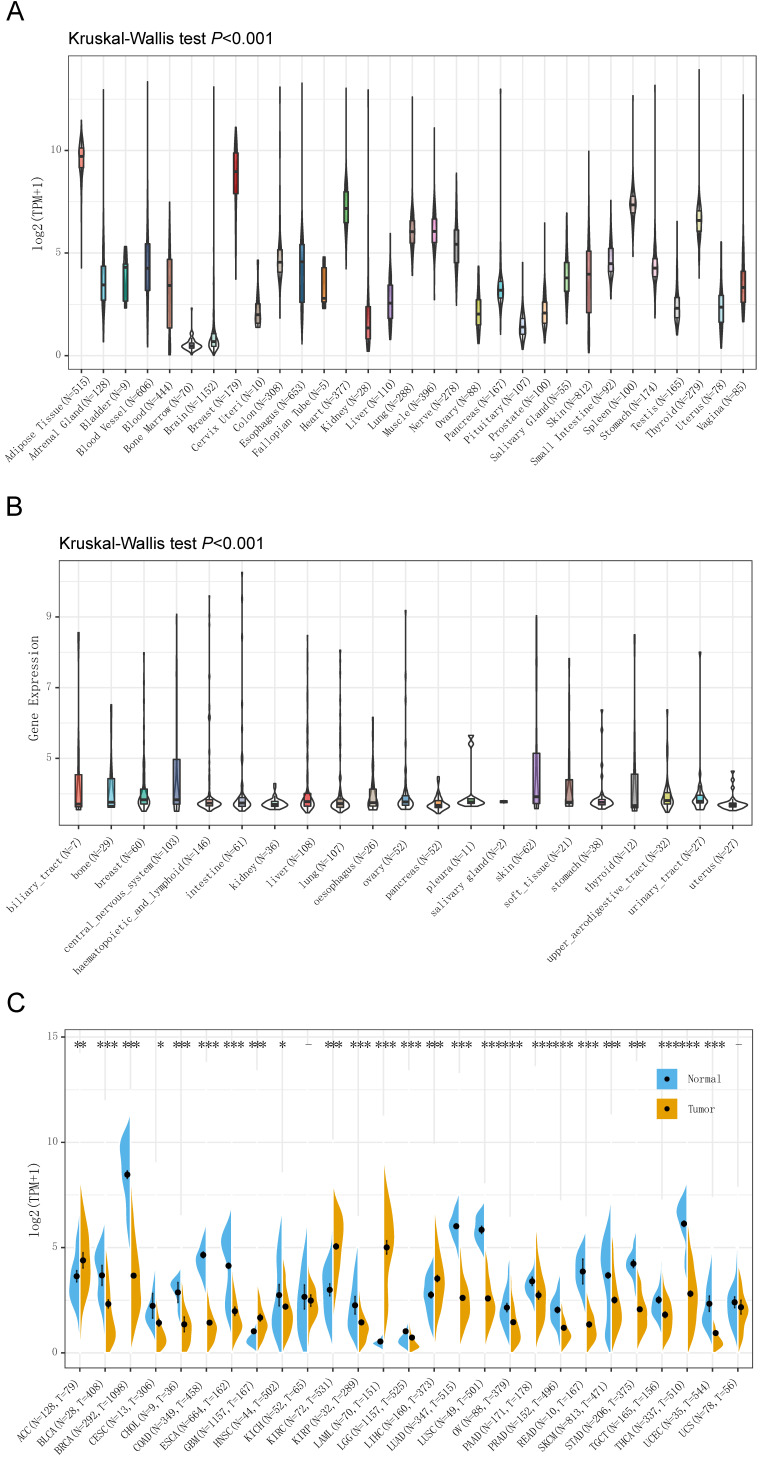
** CD36 expression in different types of tissue or cancer.** CD36 expression in (A) 31 types of tissue; (B) 21 types of cancer cell lines; and (C) 27 types of cancer. TPM, Transcripts Per Kilobase of exon model per Million mapped reads. *p<0.05, **p<0.01, ***p<0.001.

The Genotype-Tissue Expression (GTEx) database was used to analyze the gene expression in 7,858 samples from healthy individuals, and the expression of CD36 was significantly different in 31 types of tissue (Figure 1A). CD36 expression was highest in breast tissue and lowest in bone marrow.

For further analysis, we downloaded the data of 1,019 samples from cancer cell lines from the CCLE database and analyzed the CD 36 expression levels of 21 types of cancer cell lines (Figure 1B). As a result, the expression of CD36 presented high heterogeneity among most of the cancer cell lines, which might reflect its influence on the malignant behaviors of cancer cells.

The differences in CD36 expression in tumor tissue and adjacent normal tissues also displayed the heterogeneity among different cancers illustrated by the RNA-seq data of 8,624 samples in TCGA and GTEx databases (Figure 1C). CD36 expression was significantly lower in bladder urothelial carcinoma (BLCA), BRCA, cervical squamous cell carcinoma and endocervical adenocarcinoma (CESC), cholangiocarcinoma (CHOL), COAD, ESCA, head and neck squamous cell carcinoma (HNSC), kidney renal papillary cell carcinoma (KIRP), brain lower grade glioma (LGG), LUAD, LUSC, ovarian serous cystadenocarcinoma (OV), pancreatic adenocarcinoma (PAAD), prostate adenocarcinoma (PRAD), rectum adenocarcinoma (READ), skin cutaneous melanoma (SKCM), stomach adenocarcinoma (STAD), testicular germ cell carcinoma (TGCT), THCA, uterine corpus endometrial carcinoma (UCEC) than in adjacent normal tissues. However, CD36 expression was significantly higher in adrenocortical carcinoma (ACC), glioblastoma multiforme (GBM), KIRC, acute myeloid leukemia (LAML), and LIHC than in adjacent normal tissues.

**Figure 2:**
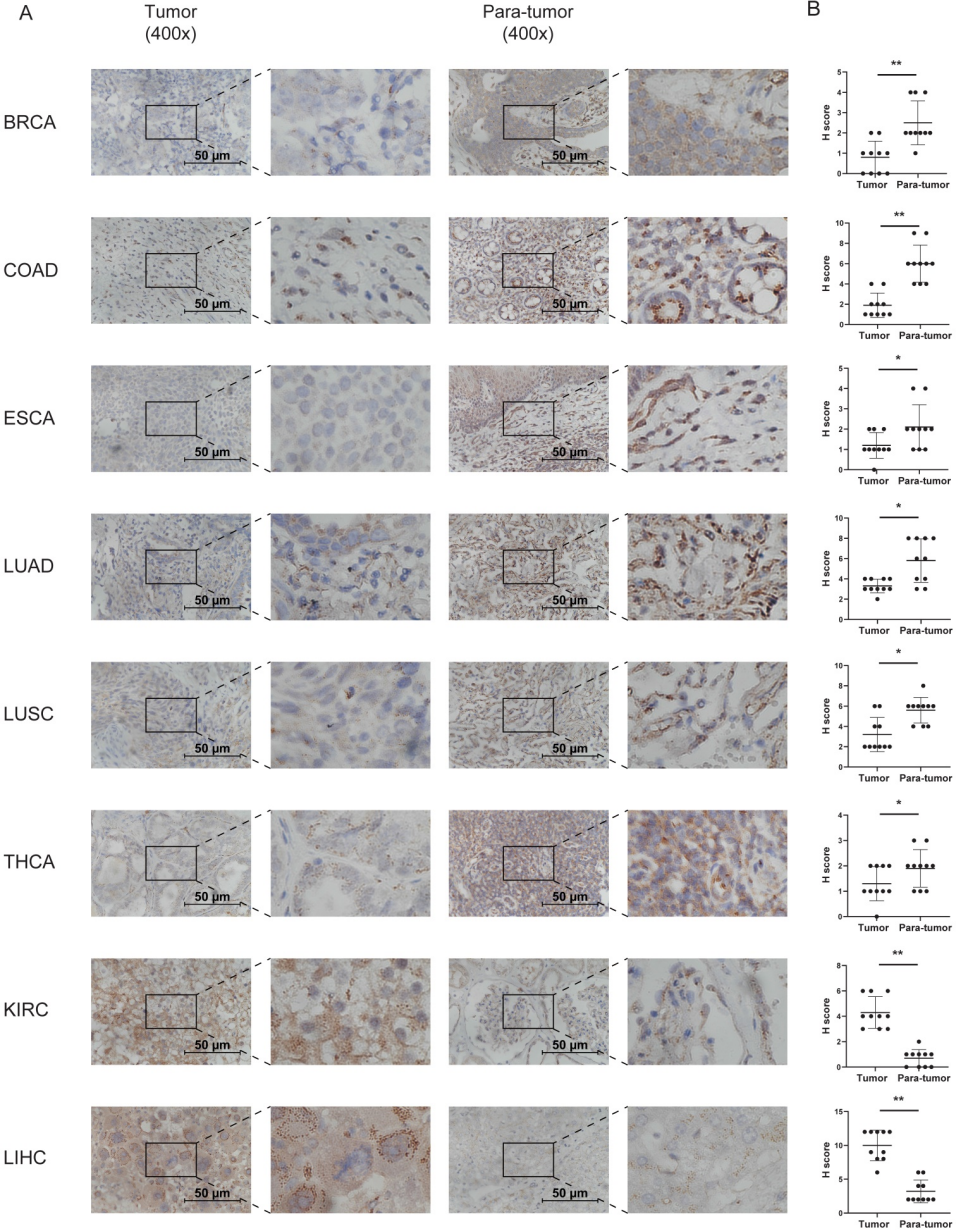
** Immunohistochemistry analysis of the expression of CD36 in tumor tissues.** (A) Typical results of one pair of samples; (B) statistical analysis. *p<0.05, **p<0.01, ***p<0.001.

Immunohistochemistry to detect CD36 expression was performed to confirm the results from TCGA and GTEx databases. CD36 protein is mainly located in the membrane of cancer cells. It was relatively higher in the tumor tissue of KIRC and LIHC and lower in BRCA, COAD, ESCA, LUAD, LUSC, and THCA (Figure 2).

### Prognostic analysis of CD36 in pan-cancer

**Figure 3:**
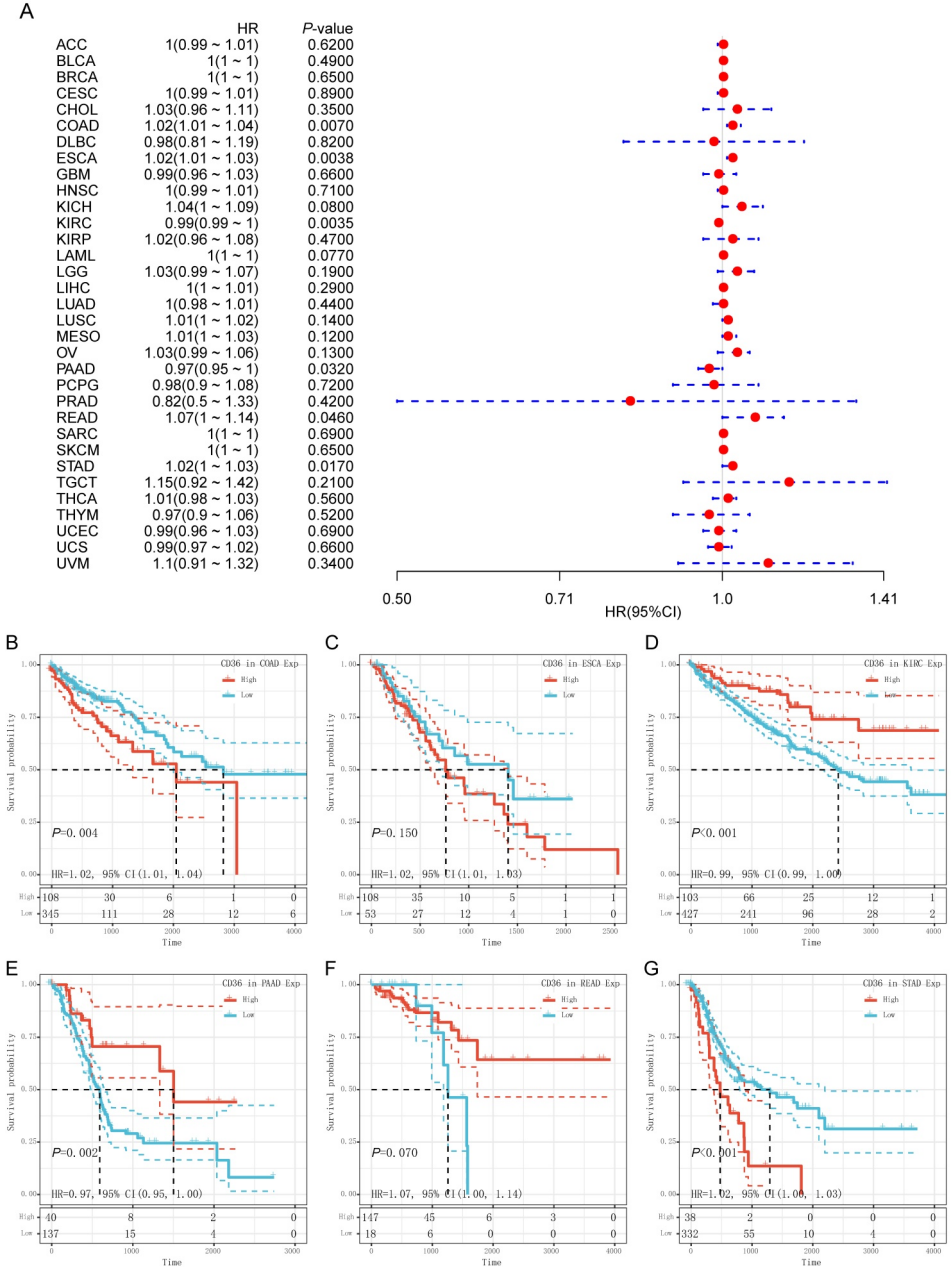
** Prognostic value of CD36 expression.** (A) Univariate Cox regression analysis of overall survival in pan-cancer. Prognostic value of CD36 in (B) colon adenocarcinoma (COAD); (C) esophageal carcinoma (ESCA); (D) kidney renal clear cell carcinoma (KIRC); (D) pancreatic adenocarcinoma (PAAD); (D) rectum adenocarcinoma (READ); and (D) stomach adenocarcinoma (STAD) by Kaplan-Meier analysis. HR, hazard ratio; CI, confidence interval.

CD36 expression presented significant prognostic value across multiple cancer types. Univariate Cox analysis was used to evaluate the association of CD36 expression with overall survival among 33 types of cancer. As shown in the forest map, CD36 expression significantly affected prognosis in COAD, ESCA, KIRC, PAAD, READ, and STAD (Figure 3A). High CD36 expression was marginally associated with poorer prognosis in four types of cancer (COAD HR = 1.02, 95% CI = 1.01 to 1.04, P = 0.007; ESCA HR = 1.02, 95% CI = 1.01 to 1.03, P = 0.004; READ HR = 1.07, 95%CI = 1.00 to 1.14, P = 0.046; STAD HR = 1.02, 95%CI = 1.00 to 1.03, P = 0.017). However, low CD36 expression was marginally associated with poorer prognosis in two types of cancer (KIRC HR =0.99, 95% CI = 0.99 to 1.00, P = 0.004; PAAD HR = 0.97, 95% CI = 0.95 to 1.00, P = 0.032). To further illustrate the prognostic potential of CD36 in different cancers, we divided patients into high and low expression groups according to the median expression. Kaplan-Meier curves confirmed the above results (Figure 3B). Above all, the expression of CD36 influenced the prognosis of multiple cancers. However, its influence on prognosis was cancer specific.

### The relationship between CD36 expression and immune infiltration

**Figure 4:**
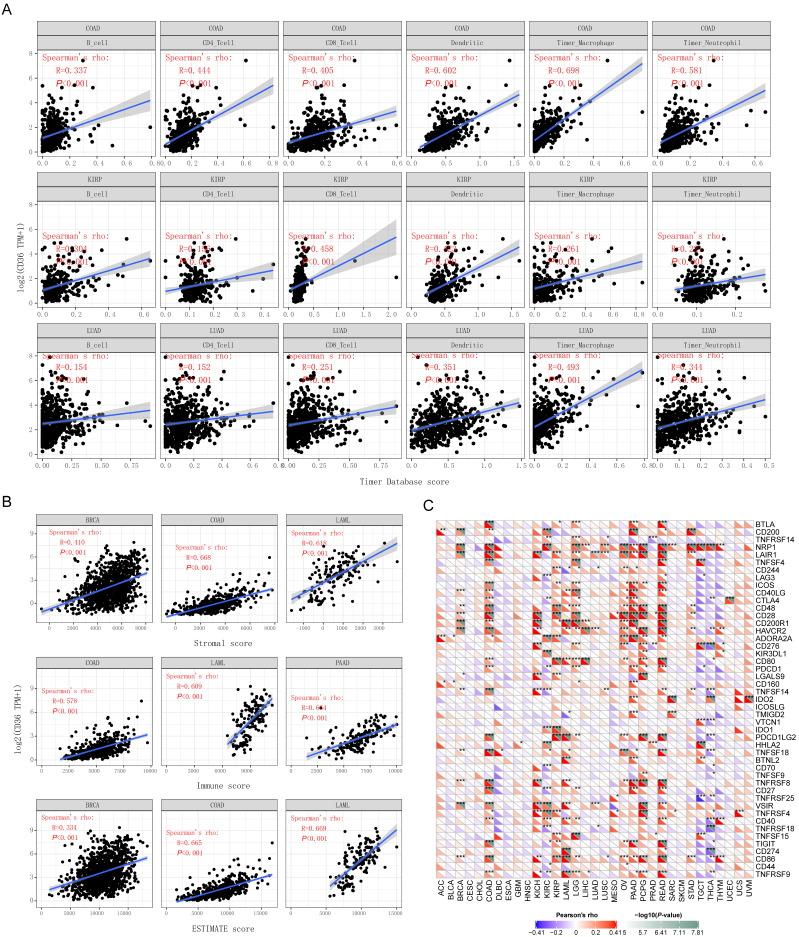
** The association between CD36 expression and immunity.** The association of CD36 expression with (A) immune infiltration, (B) immune score, and (C) immune checkpoint genes.

In addition to prognostic value, the association between CD36 alterations and 6 immune infiltrative cells (B cells, CD4+ T cells, CD8+ T cells, dendritic cells, macrophages, and neutrophils) across different cancer types was analyzed. CD36 expression was significantly correlated with the 6 immune infiltrates in most of the cancer types, with COAD, KIRP, and LUAD displaying the best correlation. CD36 expression levels were significantly and positively correlated with infiltrating levels of B cells (r = 0.337, *P* < 0.001), CD4+ T cells (r = 0.444, *P* < 0.001), CD8+ T cells (r = 0.405, *P* < 0.001), dendritic cells (r = 0.602, *P* < 0.001), macrophages (r = 0.698, *P* < 0.001), and neutrophils (r =0.581, *P* < 0.001) in COAD (Figure 4A). Similarly, there were positive correlations between CD36 expression and infiltrating levels of B cells (r = 0.304, *P* < 0.001), CD4+ T cells (r = 0.156, *P* < 0.001), CD8+ T cells (r = 0.458, *P* < 0.001), dendritic cells (r = 0.423, *P* < 0.001), macrophages (r = 0.261, *P* < 0.001), and neutrophils (r =0.221, *P* < 0.001) in KIPP. There were positive correlations between CD36 expression and infiltrating levels of B cells (r = 0.154, *P* < 0.001), CD4+ T cells (r = 0.152, *P* < 0.001), CD8+ T cells (r = 0.251, *P* < 0.001), dendritic cells (r = 0.351, *P* < 0.001), macrophages (r = 0.493, *P* < 0.001), and neutrophils (r =0.344, *P* < 0.001) in LUAD (Figure 4A). These findings suggest that CD36 plays a critical role in immune infiltration in COAD, KIRP, and LUAD 16.

### The association between CD36 expression and immune score

Stromal score, immune score, and ESTIMATE score were reported to be potential predictors for the efficacy of ICI treatment. The present study found that CD36 gene expression was positively correlated with stromal score, immune score, and ESTIMATE score (Figure 4B). CD36 expression level was significantly positively correlated with infiltrating stromal scores in BRCA (r = 0.410, *P* < 0.001), COAD (r = 0.668, *P* < 0.001), and LAML (r = 0.618, *P* < 0.001). Similarly, there were positive correlations between CD36 expression and infiltrating levels of immune score in COAD (r = 0.578, P < 0.001), LAML (r = 0.609, *P* < 0.001), and PAAD (r = 0.664, P < 0.001). There were positive correlations between CD36 expression and infiltrating levels of the ESTIMATE score in BRCA (r = 0.334, *P* < 0.001), COAD (r = 0.665, *P* < 0.001), and LAML (r = 0.669, *P* < 0.001). These findings suggest that CD36 is correlated with the tumor immune microenvironment.

### Correlation analysis between CD36 and immune checkpoint molecules

A total of 47 immune checkpoint genes were collected and the relationship with CD36 expression analyzed (Figure 4C).

CD36 expression was significantly associated with multiple stimulatory checkpoint molecules with a disease-specific pattern. Among all the significant associations, CD36 expression was positively correlated with CD27, CD28, CD40, and ICOS in multiple cancer types, but negatively associated with CD40 in TGCA (Figure 4C).

CD36 expression was significantly associated with multiple inhibitory checkpoint molecules with a disease-specific pattern. Among all the significant associations, CD36 expression was negatively correlated with ADORA2A and LAG3 in multiple cancer types, but positively associated with IDO1, IDO2, and KIR3DL1 in multiple cancers. However, the association of CD36 expression with CD274 (PDL-1) and CTLA4 was inconsistent among different cancer types (Figure 4C).

The expression of multiple genes has been reported to be positively related to the efficacy of ICIs. Among them, CD36 expression was positively correlated with CD160, CD40, CD48, CD80, CD86, CTLA4, and IDO1 but negatively associated with CD70 and LAG3 in multiple cancers. However, the association of CD36 expression with CD274 (PDL-1), TIGIT, and TNFRSF25 was cancer type-specific. CD36 expression was negatively associated with CD80, CD86, and CTLA4 in THCA and CTLA4 in KIRC. CD36 expression was positively associated with LAG3 in READ (Figure 4C).

The expression of multiple genes has been reported to be negatively related to the efficacy of ICIs. CD36 expression was positively correlated with NRP1 and TNFSF15 in multiple cancers (Figure 4C).

### CD36 expression correlates with TNB

**Figure 5:**
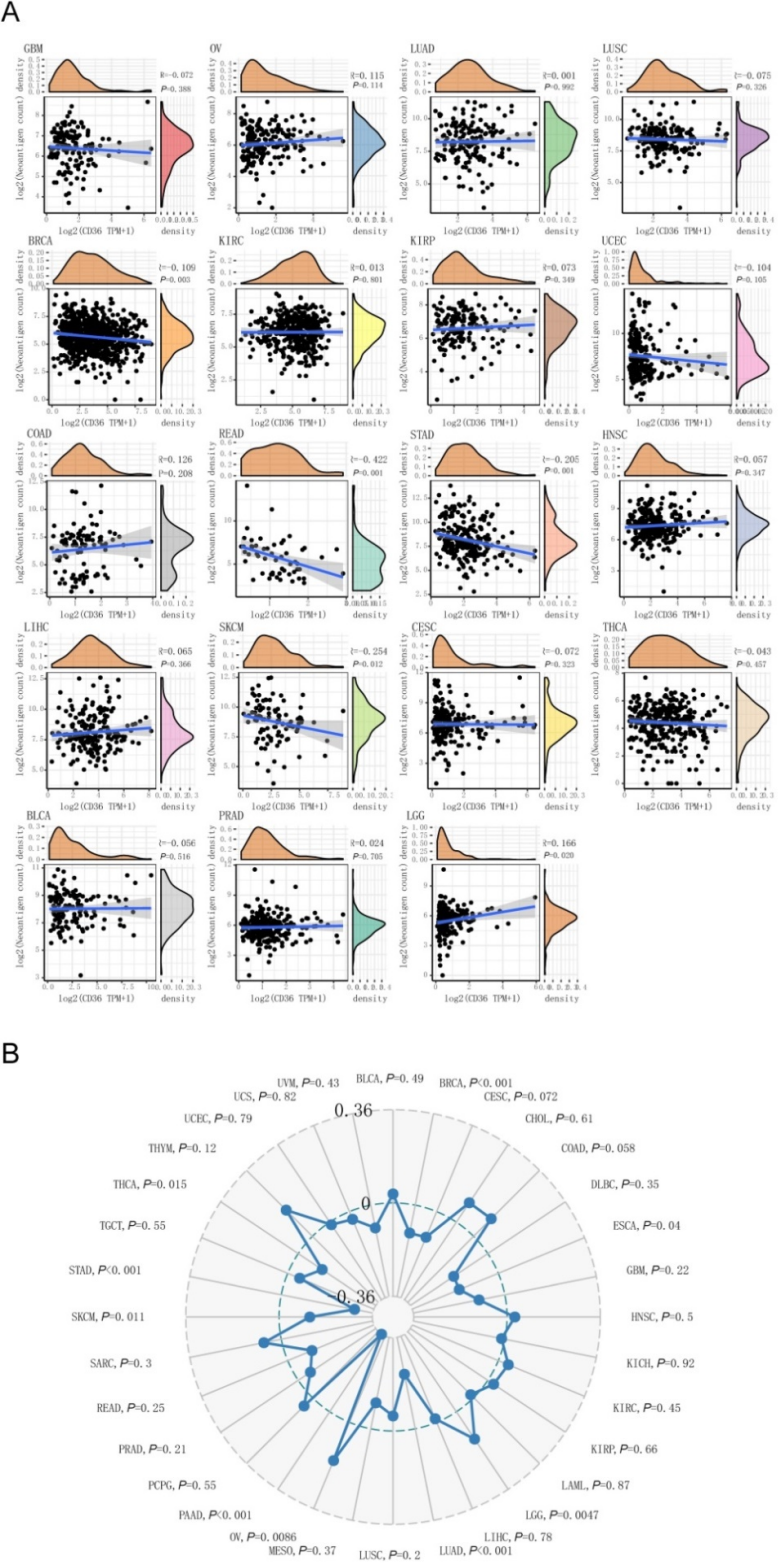
** The association between CD36 expression and neoantigen burden or tumor mutation burden.** The association of CD36 expression with (A) neoantigen burden and (B) tumor mutation burden.

CD36 was a favorable prognostic biomarker for KIRC, PAAD, and READ. Among them, CD 36 expression was negatively correlated with TNB level in READ (*P* < 0.001). CD36 expression was an unfavorable prognostic biomarker for COAD, ESCA, and STAD. Among them, CD36 expression was negatively correlated with TNB levels in STAD (*P*<0.001). In addition, CD36 expression and TNB were negatively correlated in BRCA (*P*=0.003) and SKCM (*P*=0.012), with positive correlations in LGG (*P*=0.020) (Figure 5A). Above all, CD36 expression was negatively correlated with TNB in most cancer types.

### Association between CD36 expression and TMB

CD36 was a favorable prognostic biomarker for KIRC, PAAD, and READ. Among them, CD36 expression was negatively correlated with TMB level in PAAD (*P* < 0.001). CD36 expression was an unfavorable prognostic biomarker for COAD, ESCA, and STAD. Among them, CD36 expression was negatively correlated with TMB level in ESCA (*P*=0.04) and STAD (*P*<0.001). In addition, CD36expression and TMB were negatively correlated in BRCA (*P* < 0.001), LUAD (*P* < 0.001), SKCM (*P*=0.011), and THCA (*P*=0.015), with positive correlations in LGG (*P*=0.047) and OV (*P*=0.0086) (Figure 5B). Above all, CD36 expression was negatively correlated with TMB in most cancer types 17.

### The association between the expression of CD36 and DNA methyltransferases

**Figure 6:**
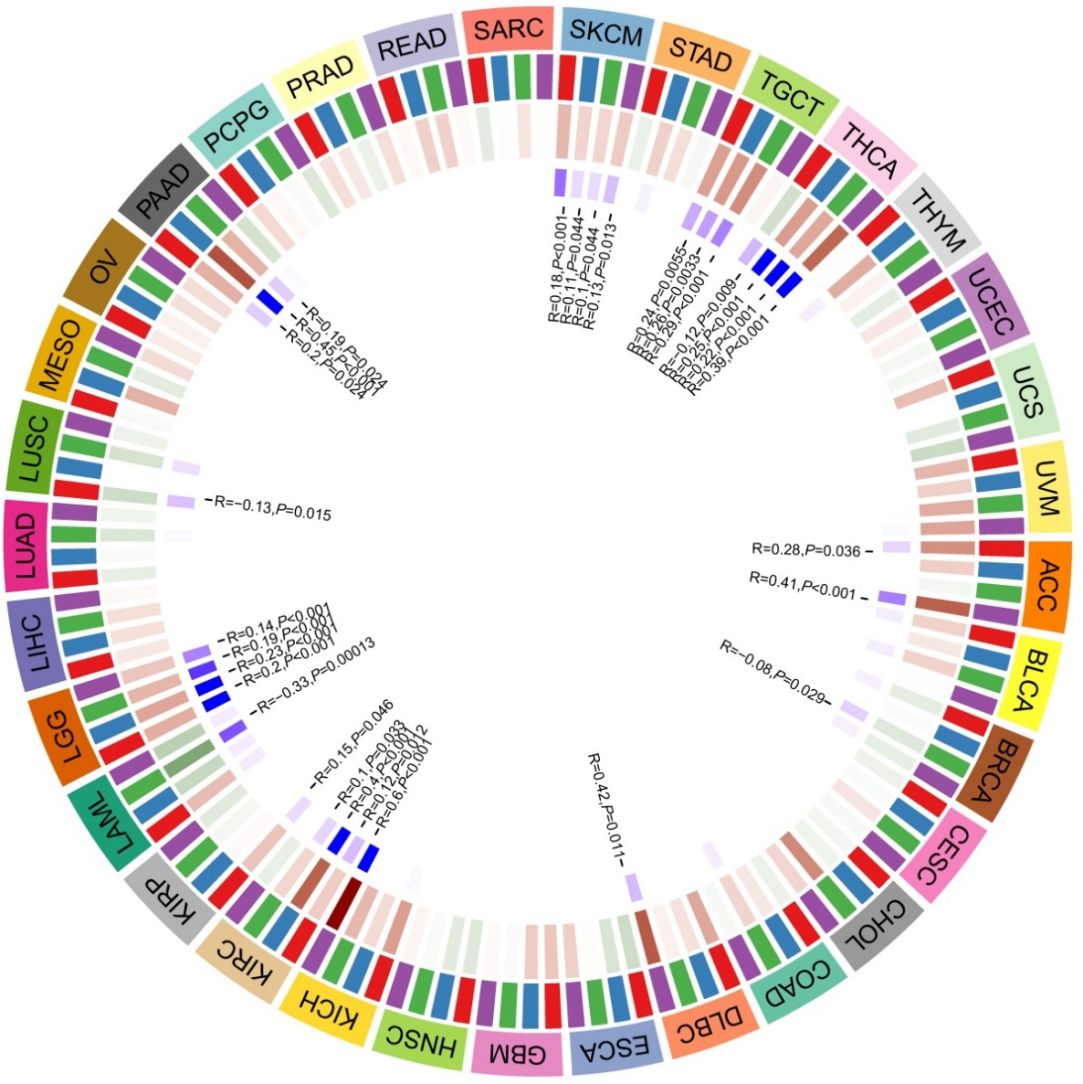
The association between CD36 expression and DNA methylation.

DNA methylation is a form of DNA modification that can change the performance of selective transmission without changing the DNA sequence. DNA methylation can cause changes in chromatin structure, DNA conformation, DNA stability, and the way DNA interacts with proteins, thereby controlling gene expression. There were positive correlations between CD36 expression and four methyltransferases in ACC, DLBC, KICH, KIRC, KIRP, LGG, PAAD, SKCM, TGCT, and THCA. There were negative correlations between CD36 expression and four methyltransferases in BRCA, LAML, and LUSC (Figure 6).

## Discussion

Multiple prognostic systems have been built for ICI treatment, including immune cells, PD-L1 overexpression, neoantigens, and genetic and epigenetic signatures 18. However, none of them achieved satisfactory accuracy. Few biomarkers predicted the efficacy of ICIs as accurate as EGFR mutations in predicting the efficacy of TKIs in non-small cell lung cancer. The predictors for the efficacy of ICIs tended to be cancer type non-specific. PD-L1 expression and dMMR/MSI status were stratification factors or patient inclusion criteria of clinical trials and indication of ICI usage in certain cancer types in treatment guidelines. However, clinical trials also found efficacy of ICIs in patients without PD-L1 expression or dMMR/MSI status 19, 20. Thus, it is imperative to optimize the prognostic system for the efficacy of ICIs among different cancer types. The present study found CD36 expression and its prognostic value in multiple cancer types. In addition, the expression of CD36 was significantly associated with current predictors for the efficacy of ICIs 21, 22.

The presence of Tumor Infiltrating Lymphocytes (TILs) in various malignancies can be used as potent predictive biomarkers for the efficacy of ICIs 23, 24. The methodology for evaluating TILs has varied among different studies. Immunohistochemical detection, RNA sequencing, and flow cytometry have been used with different immune cell types identified as potential predictors. The present study investigated the tumor infiltration of immune cells using the RNA sequence data from the TIMER database 13. We found a positive relationship between CD36 alterations and 6 immune infiltrative cells (B cells, CD4+ T cells, CD8+ T cells, dendritic cells, macrophages, and neutrophils) and immune score across different cancer types, which indicated its role in optimizing tumor infiltrate-based predictive systems for the efficacy of ICIs.

CD36 expression was negatively correlated with TMB and TNB in most cancer types. TMB is usually measured by the number of somatic mutations that occur at an average of 1 Mb in the coding region (exon region) of the tumor cell genome (non-synonymous mutations), sometimes directly. The total number of synonymous mutations indicates that the mutation types mainly include single nucleotide mutation (SNV) and small fragment insertion/deletion (Indel) and other forms of mutation. TMB is used to reflect the number of mutations contained in tumor cells and is a quantifiable biomarker 25, 26. TNB is encoded by a mutant gene of a tumor cell. It is mainly a new abnormal protein that is different from the protein expressed by normal cells and is produced by gene point mutation, deletion clip mutation, gene fusion, etc. The peptide fragments formed after enzymatic hydrolysis are presented to T cells as antigens through DC cells, which can promote the T cells to become mature activated T cells that specifically recognize the tumescent new antigen and proliferate the number of these activated T cells. The immune activity of tumor neoantigens can be used to design and synthesize a neoantigen vaccine according to the condition of the bulge of the swelling cell and immunize the patient to achieve the therapeutic effect. TNB and TMB reflect the mutation status of cancers 17, 27. The current study found that CD36 expression was negatively related to them, which indicated that CD36 might be an unfavorable predictor for the efficacy of ICIs.

The expression of multiple genes detected at the mRNA or protein level has been reported to predict the efficacy of ICIs. PD-L1 expression is the most used marker to indicate the efficacy of ICIs. However, their accuracy remains controversial. In the present study, we found that CD36 expression was positively correlated with multiple stimulatory checkpoint molecules (CD27, CD28, CD40, and ICOS) and negatively related to inhibitory checkpoint molecules (ADORA2A and LAG3). However, it was also positively correlated with multiple inhibitory checkpoint molecules (IDO1, IDO2, and KIR3DL1) in multiple cancer types. Moreover, CD36 expression presented a disease-specific association with CD274 (PDL-1) and CTLA4 among different cancer types. Thus, the practical application value of CD36 is disease specific 28-30.

In summary, the current study found CD36 expression and its prognostic value in multiple cancer types. In addition, the expression of CD36 was significantly associated with current predictors for the efficacy of ICIs. The practical application value of CD36 is disease specific 31-33.

## Abbreviations

ACC: adrenocortical carcinoma
BRCA: breast invasive carcinoma
COAD: colon adenocarcinoma
DLBC: lymphoid neoplasm diffuse large B-cell lymphoma
ESCA: esophageal carcinoma
KICH: kidney chromophobe
KIRC: kidney renal clear cell carcinoma
KIRP: kidney renal papillary cell carcinoma
LAML: acute myeloid leukemia
LGG: brain lower grade glioma
LIHC: liver hepatocellular carcinoma
LUAD: lung adenocarcinoma
LUSC: lung squamous cell carcinoma
MESO: mesothelioma
OV: ovarian serous cystadenocarcinoma
PAAD: pancreatic adenocarcinoma
PCPG: pheochromocytoma and paraganglioma
READ: rectum adenocarcinoma
SKCM: skin cutaneous melanoma
STAD: stomach adenocarcinoma
TGCT: testicular germ cell tumors
THCA: thyroid carcinoma
THYM: thymoma
UCS: uterine carcinosarcoma
UVM: uveal melanoma
TNB: tumor neoantigen burden
TMB: tumor mutational burden
MSI: microsatellite instability
MMRs: DNA mismatch repair system
